# The role of brain gaseous neurotransmitters in anxiety

**DOI:** 10.1007/s43440-021-00242-2

**Published:** 2021-03-13

**Authors:** Artur Pałasz, Itiana Castro Menezes, John J. Worthington

**Affiliations:** 1 Department of Histology, School of Medical Sciences in Katowice, Medical University of Silesia, ul. Medyków 18, 40-752 Katowice, Poland; 2 Department of Neurosciences and Behavior, Faculty of Medicine, University of São Paulo, Av. Bandeirantes 3900, Ribeirão Preto, São Paulo 14049-900 Brazil; 3 Division of Biomedical and Life Sciences, Faculty of Health and Medicine, Lancaster University, Lancaster, LA1 4YQ UK

**Keywords:** Nitric oxide, Hydrogen sulfide, Carbon monoxide, Anxiety

## Abstract

Although anxiety is perhaps one of the most significant current medical and social problems, the neurochemical mechanistic background of this common condition remains to be fully understood. Multifunctional regulatory gasotransmitters are novel, atypical inorganic factors of the brain that are involved in the mechanisms of anxiety responses. Nitric oxide (NO) signaling shows ambiguous action in animal models of anxiety, while NO donors exert anxiogenic or anxiolytic effect depending on their chemical structure, dose, treatment schedule and gas release rapidity. The majority of NO synthase inhibitors act as a relatively potent axiolytic agents, while hydrogen sulfide (H_2_S) and carbon monoxide (CO) delivered experimentally in the form of “slow” or “fast” releasing donors have recently been considered as anxiolytic neurotransmitters. In this comprehensive review we critically summarize the literature regarding the intriguing roles of NO, H_2_S and CO in the neuromolecular mechanisms of anxiety in the context of their putative, yet promising therapeutic application. A possible mechanism of gasotransmitter action at the level of anxiety-related synaptic transmission is also presented. Brain gasesous neuromediators urgently require further wide ranging studies to clarify their potential value for the current neuropharmacology of anxiety disorders.

## Introduction

Anxiety disorders, as the most common mental dysfunctions, are a serious and growing medical and social problem [1]. Despite thousands of investigative studies, the complex nature of the neurochemical basis of anxiety is still not fully understood. The fundamental roles of anxiety acquisition, its origin and manifestations involve specific neural circuits of the amygdala, dorsolateral prefrontal cingulate cortex (DLPFC), ventromedial anterior cortex and hippocampal formation [2, 3]. A wide range of neurotransmitters and neuromodulators have been demonstrated to be involved in the pathogenesis of anxiety disorder [4]. The key inhibitory neurotransmitter of the brain, g-amino butyric acid (GABA), has long been regarded as the most important factor in the etiology of anxiety, with GABA receptors being the main targets of anxiety related therapeutics such as benzodiazepines [5]. Stimulation of the GABA_A_ receptor has been associated with anxiolytic activity, whereas its inhibition may trigger anxiety responses. The expression of several enzymes responsible for neural steroidogenesis, such as 5α-reductase, 3α-hydroxysteroid dehydrogenase and aromatase (3α-HSD), have been detected in the limbic structures [6, 7], and an important role of glutamatergic neurotransmission at the level of hippocampal and amygdalar networks is widely postulated [8–10]. Serotonin and dopamine signaling, especially within the raphe nuclei, *locus coeruleus*, hippocampus and amygdala, are shown to be positively involved in the pathogenesis of anxiety [11–13]. Noteworthy, cholecystokinin (CCK)‐immunoreactive circuits and CCK2 receptors within brainstem centers may play a role in anxiety responses [14]. Neuropeptide S (NPS), urocortins and the newly identified regulatory neuropeptides nesfatin-1 and phoexinin are recently considered as potent anxiolytic factors [15, 16]. Also, adenosine acting through A1 and A2A receptors may exert anxiolysis via stimulation of GABA exocytosis in hippocampal formation [17].

The widely studied multifunctional gaseous neurotransmitters: nitric oxide (NO), hydrogen sulfide (H_2_S) and carbon monoxide (CO) are small regulatory molecules of the brain that offer a novel interest. These inorganic neuromodulators are thought to be new factors involved in the mechanisms of anxiety pathogenesis. It is interesting to postulate that the famous eighteenth century discoverers of these harmful gases Joseph Priestley (1772, NO), Carl W. Scheele (1777, H_2_S) and William Cruickshank (1800, CO) could envision a future in which these gases would be considered important molecules which play a key role in several brain functions. NO, CO and H_2_S are commonly grouped as a family of signaling molecules called gasotransmitters [18]. They have specific cellular and molecular targets and are involved in signal transduction and modulation of metabolic systems, in both physiological and pathological conditions [19, 20]. These molecules are strongly lipophilic and easily soluble, having low molecular weight, short half-life, and easily able to cross cell membranes. Different to traditional neurotransmitters, gasotransmitters are synthetized only when required, importantly avoiding the necessity of vesicle storage. They can also be released from any part of the neural or glial cell and transmission can occur in non-classical directions, for example from the postsynaptic to the presynaptic neuron or to other nearby postsynaptic cells [18, 21]. All known gasotransmitters are able to diffuse readily through the cellular lipid bilayer and do not require typical membrane receptor binding or synaptic mechanisms to initiate signaling. Just like classical neurotransmitters the effects of endogenously synthesized NO, H_2_S and CO can be mimicked by the same compounds or via their specific donors administered experimentally. All three have well defined targets in neurons or glial cells and usually act via activation of a secondary messenger (cGMP, cAMP) cascade [22, 23]. Importantly, NO, H_2_S and CO have an interdependence concerning both their own availability and that of O_2_ [18, 24]. Therefore, it is not impossible that other inorganic gas molecules such as nitrous oxide (N_2_O), sulfur dioxide (SO_2_), carbon suboxide (C_3_O_2_) [25–30] or even hydrogen cyanide (HCN) [31] can play a role of endogenous neuromodulators in some brain structures.

Currently, we have witnessed an increase in anxiety disorders hand in hand with efforts to produce more effective medications than the traditional benzodiazepines, selective serotonin reuptake inhibitors (SSRI) and 5-HT1A partial agonists. The ideal drug would only reduce anxiety symptoms with limited common side effects of the aforementioned classical anxiolytics such as dependence and withdrawal, severe sedation, memory deficits or metabolic disturbances [32–34]. Alternative treatment strategies based on modulation of other signaling pathways e.g., nitrergic and H_2_S and CO-related transmission are worth researching. Several recent studies on gasotransmitter donors and new compunds targeting NOS have shown quite promising results in animal models. Gasotransmitters are currently considered as new intriguing factors in the pathophysiology of neuropsychiatric dysfunctions including anxiety disorders. Forthcoming innovation of anxiety pharmacotherapy could potentially be based on the precise and selective modulation of NO, H_2_S and CO signaling. Therefore, wide ranging basic investigations are urgently needed to clarify the efficacy and safety of these promising treatment strategies.

## Nitric oxide in the CNS

Nitric oxide (NO) is a versatile messenger of both the central and peripheral nervous system which plays an important regulatory role in several neurochemical processes [32–34]. Brain-derived NO acts as a regulator of key functional events such as classical neurotransmitter release (e.g., serotonin, glutamate and GABA), adult neurogenesis, synaptic plasticity, neuroinflammation, cellular immunity, vascular tone, and behavioral modulation [34–39]. NO can also modulate the synthesis and release of some multifunctional neuromodulators e.g., adenosine and ATP [40]. Activation of postsynaptic glutamatergic NMDA and AMPA receptors causes postsynaptic density protein 95 (PSD95)-related stimulation of neuronal nitric oxide synthase (nNOS). This key enzyme may also interact with the C-Terminal PDZ Domain Ligand of Neuronal NO Synthase (CAPON) and Dexamethasone-induced Ras-related protein 1 (DexRas1) to trigger MAP kinase signaling (MAPK). A blockage of nNOS-CAPON signaling transmission in mice with selective disruptors ZLc-002 or Tat-CAPON12C reversed chronic mild stress-induced anxiety behavior in elevated plus maze (EPM), open field (OF) and light–dark (LD) tests. Interestingly, this kind of inhibition increased synaptogenesis and dendritic rearrangement in cultured neurons isolated from the hippocampi of stressed animals probably via the cAMP response element-binding protein (CREB)-related pathway [41]. NO molecules cross the synaptic cleft and enter into the presynaptic neuroplasm where it activates soluble guanylyl cyclase (sGC) to produce cyclic cGMP (cGMP), which is able to affect neurotransmitter release machinery and to modulate the opening of ion channels: potassium ATP-gated (K_ATP_), potassium voltage-gated (VDKC) and cationic cyclic nucleotide-gated (CNG). From the structural viewpoint GC-coupled NO receptors are heterodimers made up of a common β1-subunit together with either an α1- or α2-subunit. Importantly, the α2-subunit contains a domain that allows binding of the α2β1 isoform to protein PDZ and finally targeting it to the synaptic regions [42]. Importantly, a stimulation of cGMP synthesis may exert several anxiolytic effects and also extend benzodiazepine activity in animals (Fig. 2). NO binds to the heme group of the GC and most of the known effects of NO are due to activation of GC and the production of guanosine 3′,5′-cyclic monophosphate (cGMP), however there are several brain signaling events independent of GC activation [43]. One important alternative is S-nitrosylation of thiol groups of several neural proteins [44]. NO can also bind oxygen to form dinitrogen trioxide (N_2_O_3_) which may attact thiol groups in the proces called nitrosation [45].

It was recently reported that inosine monophosphate (IMP), which is a precursor of inosine, decreases nNOS levels in the rat cerebellum and ventral but not dorsal hippocampus. The phosphorylation of the cAMP response element-binding protein (CREB) was also increased in the ventral hippocampus and negatively correlated with nNOS expression, yet no changes in the neocortical nNOS activity were detected [46]. To conclude, NO acts as a multifunctional neuromodulatory molecule that can affect a number of signaling pathways which are involved in the mechanism of anxiety [47]. It should be emphasized that highly elevated NO concentrations may often exert diverse neurotoxic or even neurodegenerative effects such as oxidative stress reactions and induction of the apoptotic cascade [48]. An overexpression of NOS and excess of NO production may trigger neuroinflammatory processes that affect neurotransmission and other anxiety-related aspects of neuronal functions. Recent reports show that oxidative injuries are often caused by the generation of peroxynitrite (ONOO) from superoxide radicals [49, 50].

## NO synthesis and metabolism

Nitric oxide is produced via l-arginine by nitric oxide synthase (NOS), which exists in three distinct isoforms: neuronal n*NOS* (*NOS* 1), inducible i*NOS* (*NOS* 2), and endothelial *e*NOS (NOS 3). nNOS and eNOS are regulated by intracellular calcium levels (iCa^2+^) but iNOS is considered a calcium-independent enzyme. A recent study suggests interstingly, that the short allele of a functional promotor polymorphism of *NOS1* (*NOS1* ex1f-VNTR) may be associated with higher anxiety and altered fear conditioning in the human amygdala and hippocampus [51]. Several experimentally applied NOSs inhibitors are currently known, such as *N*(*ω*)-nitro-l-arginine-methyl ester (l-NAME), NG-methyl-l-arginine acetate (l-NMMA)—non selective, preferentially eNOS blockers, *N*^6^-(1-iminoethyl)-l-lysine (l-NIL) selective iNOS inhibitor, *N*(*ω*)-propyl-l-arginine (l-NPA) and 3-bromo-7-nitroindazole (3-Br-7-NI)—highly selective nNOS blockers. The initial step in the neural biotransformation of this gasotransmitter is a synthesis of *S*-nitrosothiols via binding NO molecules to activated thiols. However, nitrites and nitrates are the main oxidative metabolites of NO and the ratio of both compounds seems to be balanced by tissue redox status and therefore plasma nitrite concentration may reflect local eNOS activity and endothelial NO formation; while nitrate levels allow estimates of general N_2_/NO turnover [52]. At present numerous NO donors both classical, such as sodium nitroprusside, and new, e.g., several synthetic compounds (diazeniumdiolates, *S*-nitrosothiols) often caged inside metallic/silica or lipid nanoparticles (dendrimers and micelles) are considered to be the promising and innovative agents in experimental cancer treatment [53, 54].

## l-Ariginine: a precursor of NO biosynthesis and anxiety

Short term (4 days) oral l-arginine (Arg) and l-lysine (Lys) administration at doses of 200 mg/kg (twice daily) reduced anxiety in male rats subjected to restraint stress immediately after treatment, while plasma corticosterone levels following the elevated plus maze (EPM) test was also significantly decreased [55]. Noteworthy, a similar pattern of anxiolytic changes including reduced plasma cortisol levels has also been observed in stressed pigs fed with an Ars/Lys fortified diet [56]. An effect of Arg/Lys dietary suplementantion on anxiety states was also studied in humans, with a week long treatment with aminoacids (at dose 2.6 g/day) decreasing stress-induced anxiety, reducing concentrations of salivary chromogranin-A (a marker of adrenal sympathetic activity) and cortisol in Japanese male participants [57]. These results are in line with a previous report showing anxiolytic effects of 10 days-long Arg/Lys treatment in healthy subjects with relatively elevated trait anxiety exposed to a psychosocial stressor in the form of a public speech [58]. It is worth noting that in all aforementioned studies neither NO levels nor NOS activity were estimated. Thus, although Arg is the main precursor molecule in the NO synthesis there is no direct proof that the axiolytic action of this amino acid is a result of incresed gasotransmitter action. On the other hand, mice treated with l-arginine did not show any behavioral alterations in the EPM test but administration of this amino acid in combination with sildenafil, a phosphodiesterase 5 inhibitor, caused a significant anxiogenic effect probably through the activation of a NO-cGMP signaling pathway [59].

## Exogenous NO donors and anxiety

It should be emphasized that the number of reports dealing with the effects of NO donors in behavioral animal models of anxiety are often contradictory or ambiguous (Table 1). An injection of NOC-9, a NO donor, into mice bed nucleus of the stria terminalis (BNST), a limbic structure with abundant expression of glutamatergic, corticotrophin releasing factor (CRFergic) and nitrergic perikarya, resulted in anxiogenic effects in the EPM test. Interestingly, a previous intra-BNST injections of CP376395, a CRF type 1 receptor antagonist (CRF1), or AP-7, an NMDA (*N*-methyl-d-aspartate) attenuated the anxiety promoting effects of administered NO-donor, suggesting that CRF1 and glutamatergic signaling differentially affect NO-induced aversive behavior in the mouse BNST [60]. An acute microinjection of NOC-9 (at dose 9.4–37.5 nmol) into the mouse medial prefrontal cortex (mPFC) caused anxiogenic effect evidenced in the EPM test [61]. Conversely, an intraperitoneal administration of molsidomine, a novel NO donor induced a significant anxiolytic effects in rats in the open field (OFT) and light/dark box (LD) tests, that was notably not different to that evoked by diazepam, (a GABA agonist) treatment [62]. Intracerebroventricular infusion of the next NO donor morpholinosyndnonimine (SIN-1) at doses 0.3–1 mg/kg also resulted in anxiolytic effect in mice in the LD test [63].

**Table 1:** Summary of the main studies dealing with the relationships between gaseous neurotransmitters and anxiety

Substance	Dose	Route	Species	Effect	Methodology	References
NO precursor						
l-Arg	200 mg/kg daily	p.o. for 4 days	Rat	Anxiolytic	EPM test	[55]
l-Arg	a.l. for 10 days	Arg/Lys fort diet	Pig	Anxiolytic	Inactivity time test	[56]
l-Arg	2.6 g/day	p.o. for a week	Human	Anxiolytic	STAI inventory	[57]
l-Arg	1, 5, and 10 μg/rat	Intra amyg. inj	Rat	Anxiolytic	EPM test	[90]
l-Arg	3 g/day	p.o. for 10 days	Human	Anxiolytic	STAI inventory	[58]
l-Arg	0.4, 0.8 μg/mouse	Intra hipp. inj	Mouse	Anxiogenic	EPM test	[89]
l-Arg + sildenafil	200 + 0.05–10 mg/kg	Acute, i.p	Rat	Anxiogenic	OF test	[59]
NO donors						
Molsidomine	1, 2, 4 mg/kg	Acute, i.p	Rat	Anxiolytic	LDB and OF tests	[62]
SNP	1, 3 mg/kg	Acute, i.p	Rat	Anxiolytic at 1 mg/kg	LDB test	[64]
SIN-1	0.1, 0.3, 1 mg/mouse	Acute, i.c.v	Mouse	Anxiolytic at 0.3, 1 mg/mouse	LDB test	[63]
SNP	0.1, 0.3 and 1 mg/kg	i.p. for 5 days	Rat	Anxiolytic	LDB and OF tests	[65]
NOC-9	18.7, 37.5 or 75 nmol	Intra BNST inj	Mouse	Anxiogenic	EPM test	[60]
SNP	9.3, 18.7 or 37.5 nmol	Intra PFC inj	Mouse	Anxiogenic	EPM test	[61]
SNP	1, 2 and 3 mg/kg	Acute, i.p	Mouse	Anxiogenic at 3 mg/kg	Marble burying test	[66]
NOS inhibitors						
Aminoguanidine	50, 100, 150 mg/kg	i.p. for five weeks	Rat	Anxiolytic	EPM and OF test	[74]
l-NAME	10 mg/kg	i.p. acute or for 15 days	Rat	Anxiolytic	EPM and FST test	[79]
l-NAME/7-NI	50–200 and 5–10 nmol	Intra amyg. inj	Rat	Anxiolytic	EPM and LDB test	[80]
l-NAME	200 nmol	Intra PAG inj	Rat	Anxiolytic	LDB test	[82]
l-NAME/7-NI	10 mg/kg	Acute, i.p	Rat	Anxiolytic	EPM test	[88]
l-NAME	1, 5 and, 10 μg/rat	Intra amyg. inj	Rat	Anxiolytic	EPM and FST tests	[90]
NPLA	0.04 nmol	Intra MPFC inj	Rat	Anxiolytic	EPM test	[91]
l-NOARG	20 and 40 mg/kg	Acute, i.p	Mouse	Anxiolytic	EPM test	[87]
7-NI	20–120 mg/kg	Acute, i.p	Mouse	Anxiolytic	EPM test	[87]
l-NAME	10, 25, and 50 mg/kg	Acute, s.c	Mouse	Anxiogenic	EPM and LDB tests	[78]
l-NOARG	10 mg/kg	Acute, s.c	Mouse	Anxiogenic	EPM test	[83]
l-NOARG	10 mg/kg	Acute, s.c	Mouse	Anxiogenic	EPM test	[84]
l-NOARG	30–120 mg/kg	Acute, i.p	Rat	Anxiogenic	EPM test	[85]
l-NOARG	0.5, 1, 2 or 4 μg/μl	Intra hipp. and amyg	Rat	Anxiogenic	EPM test	[86]
l-NAME	20 and 40 mg/kg	Acute, i.p	Rat	Anxiogenic	EPM test	[87]
l-NAME	40 ng/animal	Intra hipp. inj	Rat	Anxiogenic	EPM test	[89]
H_2_S donors						
Na_2_S	4, 8 and 12 mg/kg	i.p. for 8 days	Rat	Anxiolytic	OFT test	[114]
NaHS	3 and 5 mg/kg	i.p. for 11 weeks	Rat	Anxiolytic	EPM, MWM tests	[115]
NaHS	1.68 or 5.6 mg/kg	i.p. for a week	Rat/Mse	Anxiolytic	EPM and FST tests	[113]
CO donors						
Heme lysinate	600 nmol	Intra LC inj	Rat	Anxiolytic	EPM and LDB tests	[127]
CORM-2	5 mg/kg	i.p. for 10 days	Rat	Anxiolytic	EPM and LDB tests	[133]
CORM-3	4 mg/kg	i.v. (femoral vein) in TBI	Rat	Anxiolytic	EPM and OF tests	[134]
SO_2_ donors						
Na_2_SO_3_/NaHSO_3_	5, 20, 50, 100 mg/kg	Acute, i.p	Mouse	Anxiolytic	OFT test	[137]

*amyg.* Amygdala, *BNST* bed nucleus of the stria terminalis, *CORM-2* tricarbonyldichlororuthenium [II] dimer, *CORM-3* ruthenium(II) complex Ru(glycinate)Cl(CO)_3_, *EPM* elevated plus maze test, *FST* forced swimming test, *hipp.* Hippocampus, *i.p.* intra peritoneal injection, *i.v.* intravenous injection, *LC* locus coeruleus, *LDB* light–dark box test, *MPFC* medial prefrontal cortex, *L-NAME* *N*(*ω*)-nitro-l-arginine-methyl ester, *L-NOARG* NG-nitro-l-arginine, *7-NI* 7-nitroindazole, *NOC-9* 6-(2-hydroxy-1-methyl-2-nitrosohydrazino)-*N*-methyl-1-hexanamine, *NPLA* *N*-propyl-l-arginine, *OF* open field test, *PAG* periaqueductal gray, *PFC* prefrontal cortex, *p.o.* oral treatment, *s.c.* subcutaneous injection, *SIN-1* 3-morpholinosyndnoimine, *SNP* sodium nitroprusside, *STAI* state-trait anxiety

Sodium nitroprusside (SNP), an inorganic fast NO donor in the form of ruby, water soluble crystals may regulate anxiety responses in rats under condition of the light/dark box (LD) test, although this has been shown to be dose, time and treatment schedule-dependent [64]. SNP administered 30 min before testing and not infused for 30 min, induced anxiolytic-like behavior, which could not be attributed to changes in locomotor activity. While repeated application of SNP (1 and 3 mg/kg, for 5 consecutive days) did not alter rodent behavior in both LD and motor activity tests. The authors underlined that SNP presents a narrow therapeutic window and may release potentially toxic products during its decomposition [64]. Papageorgoulis et al. [65] observed that rats subchronically treated with SNP (0.1, 0.3 and 1 mg/kg) for five consecutive days, presented a decrease in anxious behavior, whereas 0.1, 0.3 and 1 mg/kg SNP, 10 min or 30 before the test, did not exert significant modification of animals behavioral paradigms. On the other hand, intraperitoneal injection of SNP (dose 1–3 mg/kg) increased anxiety-like compulsive behavior in mice [66]. A complete mechanistic explanation of all the aforementioned contradictory reports remains elusive. Possibly, anxiogenic/anxiolytic effects of NO donors are strictly dose dependent. On the other hand NO may affect release of diverse anxiety-related both excitatory and inhibitory neurotransmitters e.g., serotonin and GABA [67, 68].

## NOS inhibitors and anxiety

Several basic studies have shown that inhibition of NO synthesis might produce anxiolytic and/or antidepressant-like effects [37, 69–71]. Almost 10 years ago Karolewicz et al. [72] found that blockade of NOS decreases serotonin turnover in the mouse frontal cortex, similar to the effect of imipramine, while a more recent report [73] showed that fluoxetine downregulates expression of nNOS in the hippocampus. Beheshti et al. [74] assessed the effect of iNOS inhibition by aminoguanidine on the development of anxiety- and depression-like behaviors using elevated plus EPM, OFT, and forced swimming tests (FST) following LPS-induced inflammation in rats. The authors reported a clear relationship between oxidative stress and behavioral disturbances, with the overproduction of NO followed by increased iNOS activity and anxiety-like behavior. Aforementioned nonselective nitric oxide synthase inhibitors (l-NAME, l-NMMA and 7-NI), act by attenuating adrenocorticotropin hormone (ACTH) responses to electric shocks [75–77], further linking NO to anxiety-related symptoms. An important study be Czech et al. [78] investigated the effects of NOS inhibition with l-NAME on anxiety related behavior in mice using EPM and LD tests. A lack of differences between D-NAME (an inactive NAME isomer) and vehicle in their actions may prove that NOS blockers act in a stereospecific manner. Some dose-related decrease was observed in the EPM test however, the effect was relatively weak and rather unclear. On the other hand, LD tests showed an anxiogenic-like action of NOS blockade, suggesting anxiolytic properties of NO [78]. However, the majority of studies report anxolytic effect of NOS inhibition in animal models. For instance, NOS blockade with l-NAME strongly prevented long-term stress-induced anxiogenesis in rats in the EPM and FST tests [79], while an analogous anxiolytic effect of bilateral l-NAME and 7-NI microinjections to the rat medial amygdala (MeA) was reported [80]. Importantly, the effect of l-NAME administration was prevented by treatment with NO donor l-arginine. Also aminoguanidine (AG) and 7-NI caused anxiolytic effect in stressed but not unstressed mice under EPM and LDT tests. The level of nitrites was also elevated in stressed rodents exclusively and it was attenuated by AG but not 7-NI administration. Of note, all effects of AG were augmented by pyrrolidine-dithio-carbamate (PDTC), an inhibitor of NF-kappaB induction, in stressed animals, suggesting an involvement of NOS in stress-induced anxiogenesis [81]. It was also recently reported that NO signaling in the periaqueductal gray (PAG) nucleus may play a role in the regulation on ethanol withdrawal-induced anxiety behavior of rats. Treatment with l-NAME caused anxiolytic effects in these animals in the LD test, whereas l-arginine abolished the action of NOS inhibitor [82].

In contrast, a number of early studies reported anxiogenic-like effects NOS inhibition. For instance, injection of NG-nitro-l-arginine (l-NOARG) abolished anxiolytic activity of chlordiazepoxide [83] and nitrous oxide [84] in mice in EPM test. Acute intraperitoneal administration of l-NOARG [at doses 30–120 mg/kg)] caused anxiogenic effects in rats in the EPM test [85]. This action was not observed after 4 days-long l-NOARG treatment (at dose 3.75–60 mg/kg, twice a day). Moreover, it was blocked by i.c.v. injection of l-arginine (dose1000 nmol). A decrease in the time spent by rats in the EPM test was also reported after targeted infusion of l-NOARG to the amygdala and hippocampus [86]. The systemic administration of l-NAME (20.0 and 40.0 mg/kg) caused anxiogenic effect in unstressed control mice but 7-NI (20.0–120.0 mg/kg) and l-NOARG (20.0 and 40.0 mg/kg) induced anxiolysis in the EPM test. Importantly, in stressed animals (small platform model), the 7-NI at a dose of 20.0 mg/kg caused in turn an anxiogenic effect, other doses of this selective nNOS inhibitor, with exception of 80.0 mg/kg, as well as l-NOARG and l-NAME did not change the rat behavior. Possibly, some alterations in the brain NOS-NO pathways as well as central action of NOS inhibitors may be stress-related [87]. Stressed rats pretreated with l-NAME or 7-NI (both at doses 10 mg/kg) exhibited distinct attenuation of anxiety responses in the EPM test. Additionally, administration of this NOS blocker reversed stress-induced increase in corticosterone and NO derivatives (NO(x)) in plasma. 7-NI, but not l-NAME, reversed stress-induced NO(x) in the hypothalamic paraventricular nucleus (PVN) and locus coeruleus (LC). Collectively, this may suggest that l-NAME affects HPA axis activity, while 7-NI may exert anxiolytic-like effects via reduction of NO(x) level in several brain structures e.g., ventromedial prefrontal cortex; vMPFC [88]. Injection of l-NAME (40 ng/animal) and l-arginine (0.4 and 0.8 μg/animal) into the mouse dorsal hippocampus induced anxiogenic responses. Both substances abolished the anxiogenic effect of histamine and pyrilamine infusion (9 mg/mouse). Furthermore, intra-CA1 administration of l-NAME and l-arginine caused anxiogenic effects [89]. Nitrergic neurons of the rat basolateral amygdala (BLA) are recently considered to play an important role in stress-dependent anxiety and depression. Stress-induced anxiety measured with the EPM test was reduced by intraamygdalar infusions of l-arginine and l-NAME (1, 5, and 10 μg/rat). Noteworthy, both agents, in all doses showed the same anxiolytic effect [90]. The anxiogenic-like effect of restraint stress evaluated with EPM test was also reversed by the injection of *N*-propyl-l-arginine (NPLA) at dose 0.04 nmol into the rat prelimbic region of vMPFC [91].

The background literature also brings contrasting results dealing with the relationship between NO signaling and anxiety in animal models (Table 1). The anxiolytic, dose dependent effects of l-arginine, NOS blockers: l-NAME, 7-NI and 8-Br-cGMP (a cGMP anaologue) microinjections into the rat dorsal raphe nucleus (DRN) are reported [92]. Of note, while l-NAME and 7-NI at low doses caused anxiolytic-like effects, higher doses decreased locomotor activity. On the other hand, 8-Br-cGMP (25 and 50 nmol) increased the number of closed arm entries but did not produce changes in anxious behaviors measured with the EPM test. Dual effects on anxiety following interference with NO-mediated neurotransmission were reported [92]. Corroborating to the existing duality, Pitsikas [38] pointed out that, it was not possible to conclude whether activating NO pathways leads to increased or reduced anxiety-like behavior. Furthermore, it is also highlighted that there is no information regarding the implication of eNOS and iNOS on psychiatric disorders nor is it clarified if the effects of NO on anxiety are sex-dependent or not.

## Brain-derived hydrogen sulfide

Hydrogen sulfide (H_2_S) is commonly known as a toxic, colorless gas with the characteristic obnoxious odor of rotten egg proteins. The majority of current reports unambiguously suggest that the H_2_S molecule should be attached to the family of gaseous neurotransmitters in addition to nitric oxide and carbon monoxide [93–96]. In the CNS, H_2_S is involved in several processes facilitatiing the long-term potentiation (LTP) and upregulation of the GABA_B_ receptor in the hippocampus [97, 98]; modulation of the hypoxic ventilator response; regulation of intracellular calcium homeostasis [99], pH balance in neuronal and glial cells [99] and neuroprotection against oxidative stress [100]. Sodium hydrosulfide (NaHS) injection decreased hippocampal impairment in the rat brain after cerebral ischemia [101], but gaseous H_2_S reduced the neurological damage in the same experimental condition of common carotid artery occlusion [102]. However, it should be emphasized that in the animal model of stroke only low doses of NaHS exerted a neuroprotective effect, with higher ones being neurotoxic [103]. Furthermore, it was reported that Na_2_S may increase neuronal survival after ischemic injury of mouse cardiac muscle and animals with heart CSE overexpression had a better neurological outcome following the tape removal test [104]. Treatment with Na_2_S may also have beneficial behavioral effects in the rat model of cardiac arrest, however it does not affect neuronal apoptosis processes [105].

## Neural and glial H_2_S biosynthesis and metabolism

**Fig. 1:**
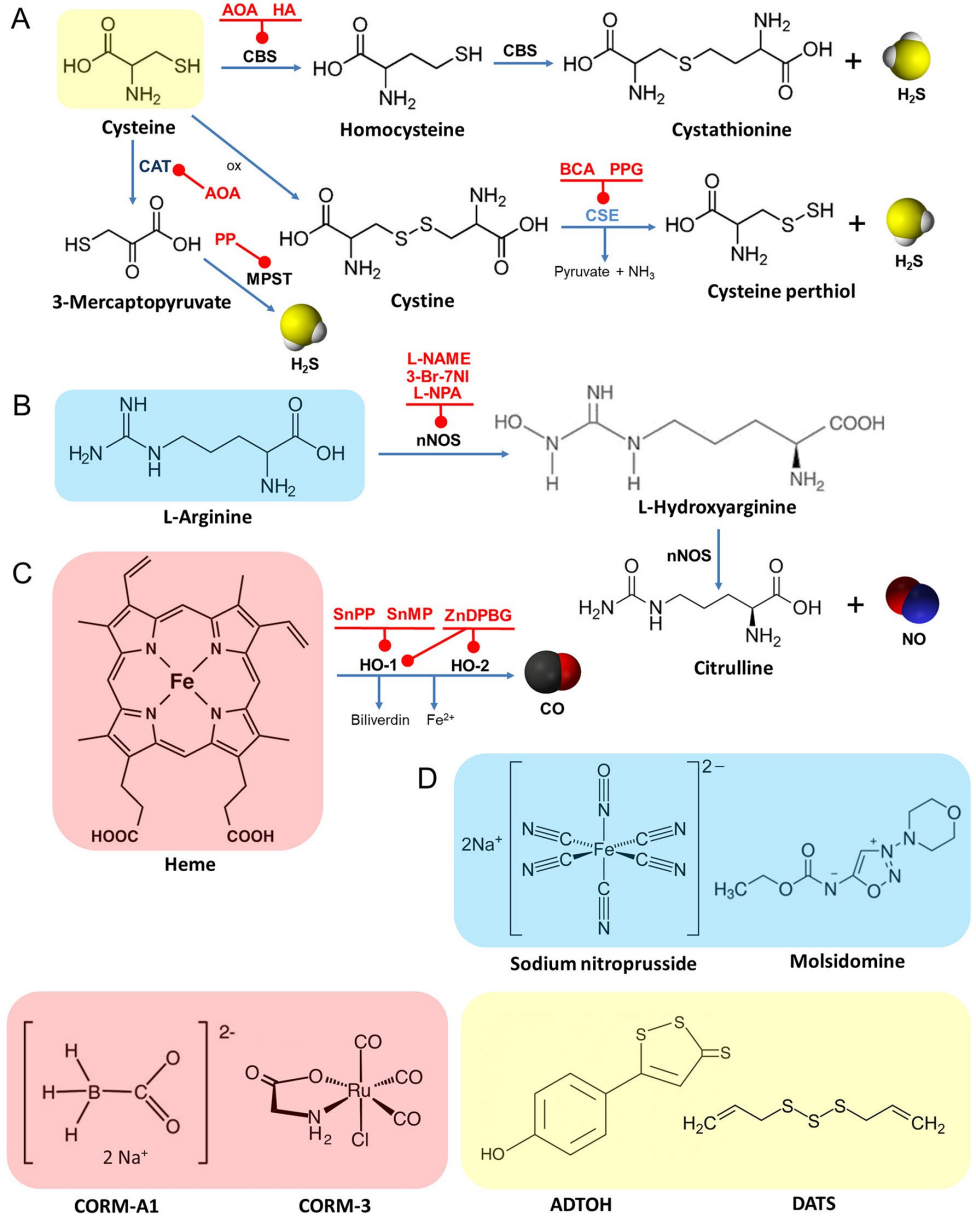
The main pathways of gasotransmitter biosynthesis in the brain with key regulatory enzymes and their exogenous inhibitors. Schematic representation outlines the origin of hydrogen sulfide (**a**), nitric oxide (**b**) and carbon monoxide (**c**) from the precursors (on different color backgrounds). All enzyme blockers are marked in red and round-ended lines represent their inhibitory effect. The molecular structures of selected exogenous donors of NO, H_2_S and CO are also shown (**d**). Anethole dithiolethione; *ADTOH* aminooxyacetate, *AOA* b-cyano-l-alanine, *BCA* 3-bromo-7-nitroindazole (3-Br-7-NI), *3-Br-7NI* cysteine aminotransferase, *CAT* cystathionine-b-synthase, *CBS* sodium boranocarbonate, *CORM-A1* tricarbonylchloro(glycinato)ruthenium, *CORM-3* cystathionine-g-lyase, *CSE* diallyl trisulfide, *DATS* hydroxylamine, *HA* heme oxygenases 1 and 2, *HO-1/HO-2* *N*(*ω*)-nitro-l-arginine-methyl ester, *L-NAME* *N*(*ω*)-propyl-l-arginine, *L-NPA* mercaptopyruvate sulfurtransferase, *MPST* phenylpyruvate, *PP* propargylglycine, *PPG* tin mesoporphyrin, *SnMP* tin protoporphyrin, *SnPP* zinc deuteroporphyrin 2,4-bis glycol; ZnDPBG

Hydrogen sulfide is synthesized in both neurons and astrocytes from l-cysteine (Cys) with the participation of three key enzymes: cystathionine b-synthase (CBS), cystathionine g-lyase (CSE) and cysteine aminotransferase (CAT), Fig. 1. Catalytic activities of CBS and CSE are tissue specific [106]. Also 3-mercaptopyruvate sulfurtransferase (MPST) is involved in brain H_2_S synthesis from 3-mercaptopyruvate (3-MPT). Of note, an interesting crosstalk between NO and CO pathways may regulate heme moiety of CBS has recently been suggested [107–109]. A non-enzymatic reaction of H_2_S synthesis which is catalyzed by pyridoxal 5′-phosphate (vitamin B6) and iron may also occur in some tissues especially within erythrocytes [110]. The metabolic elimination of H_2_S is based on its oxidation to persulfide by mitochonrdrial sulfide quinone oxidoreductase (SQR), persulfide dioxygenase (ETHE1) and also by the methylation via the cytoplasmic enzyme cysteine dioxygenase (CDO) [111]. The further oxidation of persulfide by ETHE1 results in sulfite formation and finally sulfites are metabolized by sulfite oxidase or rhodanese (thiosulfate sulfurtransferase) to rhodanide and sulfate respectively. Probably, superoxide dismutase (SOD) may oxidize H_2_S to polysulfides [112]. A number of exogenous H_2_S donors are known, such as rapid-releasing alkaline sulfides (Na_2_S, NaHS) and slow liberating sulfur compounds e.g., S-memantine, dithioloethione (ADT) and dially trisulfide (DATC) [96].

## Sodium sulfide: a fast exogenous H_2_S donor and anxiety

**Fig. 2:**
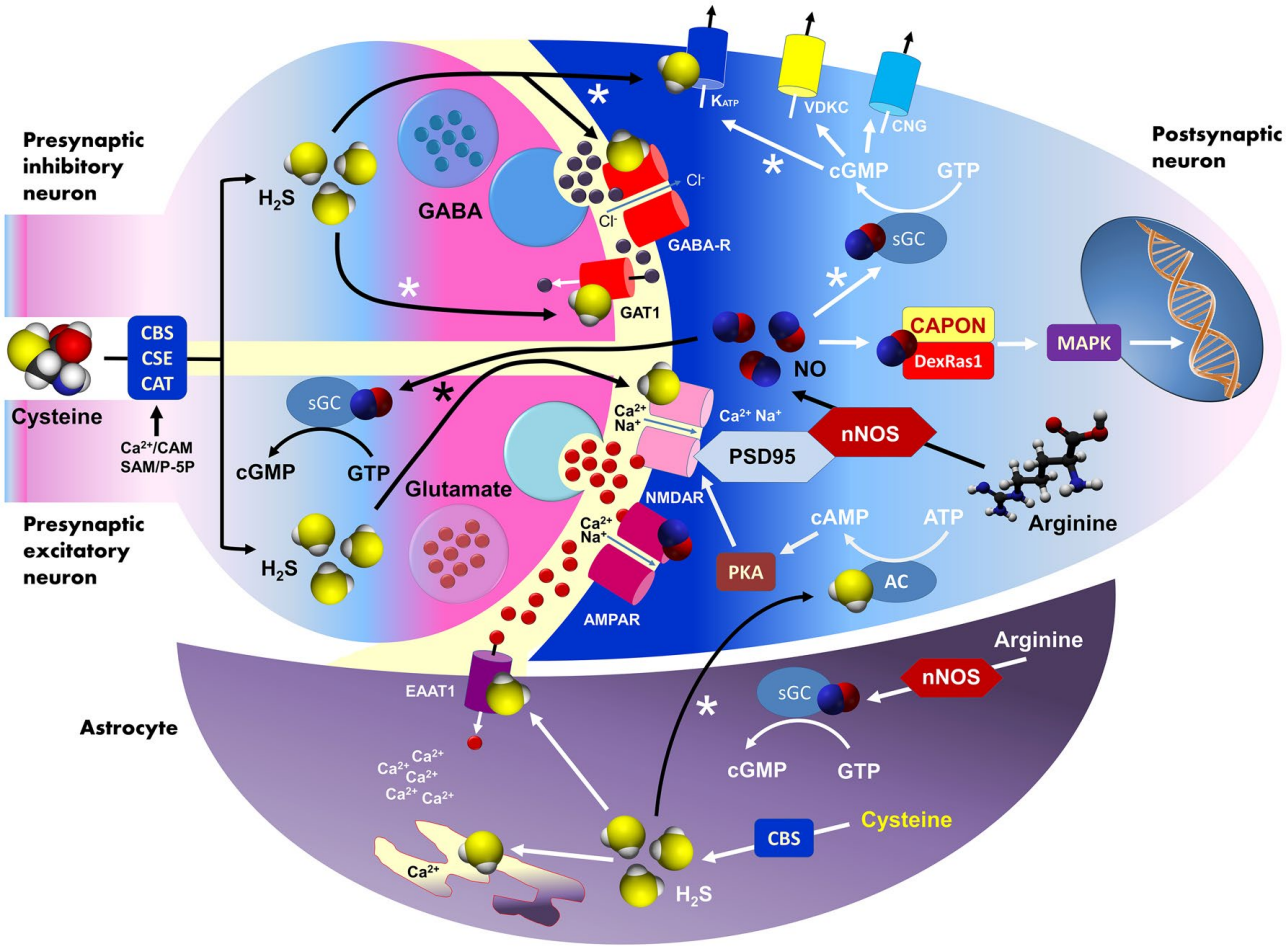
Neurochemistry of nitric oxide and hydrogen sulfide in the context of anxiety-related cellular mechanisms. All molecular events potentially responsible for anxiolytic/anxiogenic properties of NO and H_2_S were marked with asterisks. The figure is an original conceptual artwork partly based on data from selected references [37, 38, 98, 138]

Currently, some studies are investigating the effects of H_2_S in depressive- and anxious-like behaviors. Chen et al. [113] investigated the anxiolytic and antidepressant effects of NaHS (doses 1.68 or 5.6 mg/kg) in rats and mice. Applying EPM and forced swimming test (FST) protocols, they observed antidepressant and anxiolytic effects in mice, while only anxiolytic effects in rats. Significantly, either NaHS or imipramine, applied intraperitoneally for 7 days, has shown antidepressant effects when compared to saline. Likewise, Donatti et al. [114] observed anxiolytic effects of 8 day-long sodium sulfide (Na_2_S) administration (dosage 4, 8 and 12 mg/kg) in rats during OFT. Corroborating these results, Chen et al. [107] correlated the current literature highlightening the potential role of H_2_S on pathophysiology and management of anxiety disorders, and the role of H_2_S in neuroplasticity and oxidative stress regulation by decreasing reactive oxygen species. Increasing neuroplasticity would likely resulti from glutamatergic transmission via NMDA receptors, long-term potentiation, and within neuronal and glial Ca^2+^ homeostasis maintainance. A new study by Habibitabar et al. [115] reported that NaHS significantly reduced anxiety-like behavior and improved memory in rats treated with a high-fat diet (HFD). It is not yet clear how H_2_S can impact the brain mechanism of anxiety. Potentially, this may stimulate GABA-related transmission (Fig. 2) and maintain intracellular reduced brain glutathione to decrease oxidative stress. Alternatively, it may facilitate amydalar or hippocampal neuroplasticity via NMDA-dependent signaling and/or modulate neural and glial Ca^2+^ homeostasis [107]. In the case of glutamatergic neurons H_2_S activates NMDA receptor similarly to NO. On the other hand, in the inhibitory GABAergic neuron H_2_S may stimulate presynaptic GABA transporter 1 (GAT1) that increases GABA reuptake and also directly modulates poststnaptic GABAA receptors as well as K_ATP_ potassium channels. An increase in synaptic GABA concentration promotes postsynaptic neuron silencing that may support anxiolytic effects. Astrocytes are considered as alternative source of H_2_S in the brain, with the gas molecules produced activating adenylyl cyclase (AC) of adjacent neurons and modulate NMDA receptor signaling via cyclilc AMP (cAMP) signaling. H_2_S can also cause rapid calcium release from astrocytic endoplasmic reticulum and the activation of excitatory amino acid transporter (EAAT1) that increases synaptic glutamate level.

Anxiolytic effects in experimental, preclinical, and initial pharmacologic studies of H_2_S-releasing drugs such as S-diclofenac, natural sulfide compounds e.g., garlic and breathing retraining programs (which produce more available H_2_S) have also been decribed. A unique preclinical study reported intriguing functional effects of H_2_S on anxiety and fear extinction-related neurocircuits in the amygdala. Interestingly, breathing retraining programs, which are incorporated into tai chi and yoga, may increase plasmatic H_2_S, NO and CO molecules level and significantly reduce anxiety ratings in participants with hypertension [108, 116]. However, further studies are required to asses the potential applicability and innovational therapeutic actions of these techniques.

## Carbon monoxide in the brain

Carbon monoxide (CO) is commonly known as a highly toxic, haemoglobin deactivating and seriously hazardous industrial gas, a product of incomplete hydrocarbon combustion. However, CO is synthesized endogenously in several tissues where it can be involved in numerous biochemical and physiological processes such as signal transduction, blood pressure regulation, inhibition of myocytes proliferation, modulation of cell activity and maintaining of neural homeostasis and having an anti-inflammatory, anti-thrombotic and anti-apoptotic effect [22, 117]. In the brain CO activates sGC to increase cGMP stimulation of PKG. CO and NO bind sGC with similar affinity but CO-sGC is 25–50 times less active than NO-sGC [118]. CO may extend the opening time of calcium-gated large conductance potassium channels (BKCa) via binding to their heme domains [119, 120] and block inward rectifier channels (*K*_ir_) via an unknown mechanism [121]. Endogenous CO at normal temperature and pressure is chemically inert in the absence of *d*-electron metals [122].

There is also a suggestion that CO may affect synaptic turnover and signaling through the modulation of dopamine and glutamate transporters. The modulation of cGMP synthesis by CO seems to be NO independent, structure specific and even sexually dimorphic e.g., exogenous CO increased striatal cGMP levels only in male individuals [123]. Since the early 1990s, CO therapeutic effects have been investigated as potential agents for treatments of diseases such as cancer, sepsis, hypertension, heart failure, inflammation, thrombosis, neurodegeneration and hematological diseases [23, 24, 124, 125]. However, the number of reports regarding potential effects of CO and its donors on anxiety is still scarce.

The possible forms of applying CO for both experimental and therapeutic purposes are inhaled gas, and safer CO releasing molecules (CORMs) that may be administered per os or parenterally. CORMs are unique boron (CORM-A1, a “slow” CO releaser) or heavy metals (CORMs 1–3) containing carbonyl complexes that aim to deliver, in a controlled way, limited amounts of CO to diverse tissues including brain structures [23, 125, 126] offering potentially therapeutic fine tuning of CO liberation.

## CO biosynthesis

Carbon monoxide (CO) is a gasotransmitter that stimulates guanylyl cyclase (GC) to form cGMP and acts by binding to iron atom at the active site of heme [117]. The endogenous production of CO occurs through the activity of heme oxygenase (HOCO, HOs) responsible for the cleavage of heme molecule into biliverdin, free iron (Fe^2+^) and CO. Two independent isoforms of this enzyme are known: an inducible heme oxygenase 1 (HO-1) and a constitutive form, heme oxygenase 2 (HO-2) [24]. A third probably inactive isoform HO-3 was cloned from rat brain tissue but its functions remains unknown [127, 128]. The expression of HO-1 is detected in the selective small cellular assemblies of the brain (both neural and glial) while distribution of HO-2 is rather ubiquitous [129]. Importantly HO-1 activity is upregulated by cellular stress suggesting that this enzyme represents the heat-shock protein family [103]. Relatively abundant HO-2 expression has been found in the hippocampal formation, hypothalamus, neocortex, cerebellum and olfactory bulb [130, 131]. HOs activities may be unspecifically blocked by clemizole derivatives or synthestic imidazoles e.g., azalanstat, but some kinds of tin porphyrin complexes (SnPP, SnMP) are considered as selecive HO-1 inhibitors. Tissue-derived CO, too dilute to affect oxygen turnover is exhaled through the lungs [23].

## Exogenous CO donors and anxiety

One of the few pioneer studies on CO and anxiety was conducted by Carvalho-Costa et al. [127]. This important report showed that anxiolytic effect of CO in rats was evoked by cGMP-dependent heme oxygenase pathway in the rat locus coeruleus (LC), the main center of brain noradrenegic signaling [127]. Noteworthy, LC neurons show abundant HO-2/HO-1 expression suggesting a distinct role of CO-related mechanisms in their regulatory functions [132]. Targeted intra-LC microinjection of heme-lysinate (a substrate for HOs) significantly decreased anxiety symptoms during EPM and LDB tests. Conversely, microinjection of a selective sGC inhibitor (ODQ) s prior to heme-lysinate abolished all aforementioned anxiolytic effects. On the other hand a treatment with zinc deuteroporphyrin 2,4-bis glycol (ZnDPBG), a nonselective HOs blocker did not alter animal behavior [127]. All neurochemical and physiological phenomena perceived in this investigation strongly suggest that the anxiolytic action of CO within rat *locus coeruleus* is strictly connected with local cGMP signaling. The aforementioned results were supported by amore recent study showing that CO, liberated both acutely (3 h) and chronically (10 days, twice a day) from an intraperitoneally administered tricarbonyldichlororuthenium [II] dimer (CORM-2) molecule promoted an anxiolytic-like effect (examined with EPM and LD tests) and increased HO-1 activity in the rat LC [133]. Additionally, both short- and long-term treatment with cobalt protoporphyrin IX, (CoPP), a HO-1 inducing complex resulted in the same behavioral effects. Treatment with CORM-3 significantly reduced anxiety-like behavior in rats after transitional brain ischemia (TBI), improved brain circulation and promoted neuronal survival in the amygdala [134].

By applying intracerebroventricular injections of tin protoporphyrin (SnPP, selective HO-1 inhibitor) and l-NAME, Kim and Rivier [77] were able to verify that NO and CO may exert a stimulatory influence in physicoemotional stressors (ACTH mediated responses) and that the hypothalamus is a critical site for the central regulatory actions of both gasotransmitters. The authors remarked that NO (and also CO) might exert opposite effects in the hypothalamic paraventricular nucleus (PVN) when compared to other neuroendocrinal structures (median eminence and pituitary) [77]. The same research group had already found that there was a blockade of endogenous CO formation after systematic HO-1 blockers: SnPP and tin mesoporphyrin (SnMP) injections, followed by a reduction of plasmatic ACTH when rats were exposed to mild electroshocks [76].

## Sulfur dioxide and anxiety responses

Interestingly sulfur dioxide (SO_2_) synthesized during intracerebral amino acid metabolism has also been very recently suggested as a new gaseous transmitter [27]. Brain-derived SO_2_ a product of aspartate aminotransferase (AAT) catalytic activity can easily pass through all neurolemmal barriers and is considered to be involved in some cardiovascular regulatory processes including blood pressure control [135]. This novel study reported that treatment with SO_2_ significantly reduced neuronal injury in the rat hippocampus after chronic cerebral hypoperfusion (CCH) and improved cognitive disturbances in the Morris water maze. Of note, hippocampal catalase activity was also elevated suggesting that SO_2_ may support brain antioxidant responses [136]. It was recently reported that treatment with exogenous SO_2_ donors sodium sulfite and hydrosulfite (Na_2_SO_3_:NaHSO_3_) caused a distinct anxiolytic-like effect in mice (in the OFT test) under normal conditions and abolished chronic mild stress (CMS)-induced anxiety-like behavior. This may open a cautious discussion about SO_2_ as a new possible agent for the treatment of anxiety [137, 138].

## Concluding remarks

A number of reports have clearly demostrated that nitric oxide molecules are involved in the neurochemistry of anxiety responses in animal models and both NO donors and selective NOS inhibitors can be rightly considered in the future treatment of anxiety disorders. Moreover, recent findings have revealed that hydrogen sulfide and carbon monoxide may also exert distinct anxiolytic effects and its donors seem to be applicable. It should not be excluded that a novel way to treat anxiety has been postulated and may be an innovative alternative in the development of new therapeutic possibilities. However, there are still numerous concerns related to the pharmacological effects of gaseous neurotransmiter donors or modulators and further basic investigations are urgently required to prove their potential usefulness for the pharmacotherapy of anxiety. Firstly, all potential treatment schedules either acute or long-term should be precisely evaluated. Secondly, more detailed animal studies should assess the efficacy and safety-profile of NO, H_2_S and CO donors as anxioltytic factors using a large spectrum of behavioral methods. Importantly, a relatively narrow therapeutic window of these substances as well as their potential biphasic actions should also be taken into account. To conclude, an alternative way to treat anxiety has potentially been identified and it may introduce a new therapeutic strategy in current neuropsychiatry. Nevertheless, there are still numerous questions related to the anxiolytic properties of gaseous neurotransmitters and more advanced pharmacological studies are needed to confirm their possible clinical applicability.

## References

[1] Stein DJ, Scott KM, de Jonge P (2017). Epidemiology of anxiety disorders: from surveys to nosology and back. Dialogues Clin Neurosci.

[2] Myers-Schulz B, Koenigs M (2012). Functional anatomy of ventromedial prefrontal cortex: implications for mood and anxiety disorders. Mol Psychiatry.

[3] Ninan PT (1999). The functional anatomy, neurochemistry, and pharmacology of anxiety. J Clin Psychiatry.

[4] Kaur S, Singh R (2017). Role of different neurotransmitters in anxiety: a systemic review. Int J Pharm Sci Res.

[5] Lydiard RB (2003). The role of GABA in anxiety disorders. J Clin Psychiatry.

[6] Carta GM, Bhat MK, Preti MK (2012). GABAergic neuroactive steroids: a new frontier in bipolar disorders?. Behav Brain Funct.

[7] Bogus K, Pałasz A, Suszka-Świtek A, Worthington JJ, Krzystanek M, Wiaderkiewicz R (2019). Chronic antipsychotic treatment modulates aromatase (CYP19A1) expression in the male rat brain. J Mol Neurosci.

[8] Bergink V, van Megen HJ, Westenberg HG (2004). Glutamate and anxiety. Eur Neuropsychopharmacol.

[9] Garakani A, Mathew SJ, Charney DS (2006). Neurobiology of anxiety disorders and implications for treatment. Mt Sinai J Med.

[10] Millan MJ, Brocco M (2003). The Vogel conflict test: procedural aspects, gamma-aminobutyric acid, glutamate and monoamines. Eur J Pharmacol.

[11] Akimova E, Lanzenberger R, Kasper S (2009). The serotonin-1A receptor in anxiety disorders. Biol Psychiatry.

[12] Jia M, Pittman J (2014). Deficits in striatal dopamine and hippocampal serotonin following induction of anxiety/depressive like behaviors by bisphenol A. Arch Neurosci.

[13] Murphy DL, Moya PR, Fox MA, Rubenstein LM, Wendland JR, Timpano KR (2013). Anxiety and affective disorder comorbidity related to serotonin and other neurotransmitter systems: obsessive– compulsive disorder as an example of overlapping clinical and genetic heterogeneity. Philos Trans R Soc B.

[14] Chen Q, Nakajima A, Meacham C, Tang YP (2006). Elevated cholecystokininergic tone constitutes an important molecular/neuronal mechanism for the expression of anxiety in the mouse. Proc Natl Acad Sci.

[15] Grund T, Neumann ID (2019). Brain neuropeptide S: via GPCR activation to a powerful neuromodulator of socio-emotional behaviors. Cell Tissue Res.

[16] Pałasz A, Janas-Kozik M, Borrow A, Arias-Carrión O, Worthington JJ (2018). The potential role of the novel hypothalamic neuropeptides nesfatin-1, phoenixin, spexin and kisspeptin in the pathogenesis of anxiety and anorexia nervosa. Neurochem Int.

[17] Maximino C, Lima MG, Olivera KR, Picanço-Diniz DL, Herculano AM (2011). Adenosine A1, but not A2, receptor blockade increases anxiety and arousal in Zebrafish. Basic Clin Pharmacol Toxicol.

[18] Donald JA (2016). Gasotransmitter family. Handb Horm.

[19] Kajimura M, Nakanishi T, Takenouchi T, Morikawa T, Hishiki T, Yukutake Y (2012). Gas biology: tiny molecules controlling metabolic systems. Respir Physiol Neurobiol.

[20] Wang Y, Yu R, Wu L, Yang G (2020). Hydrogen sulfide signaling in regulation of cell behaviors. Nitric Oxide.

[21] Haley JE (1998). Gases as neurotransmitters. Essays Biochem.

[22] Untereiner AA, Wu L, Wang R, Hermann A, Sitdikova G, Weiger T (2012). The role of carbon monoxide as a gasotransmitter in cardiovascular and metabolic regulation. Gasotransmitters: physiology and pathophysiology.

[23] Wu L, Wang R (2005). Carbon monoxide: endogenous production, physiological functions, and pharmacological applications. Pharmacol Rev.

[24] Motterlini R, Otterbein LE (2010). The therapeutic potential of carbon monoxide. Nat Rev Drug Discov.

[25] Jevtović-Todorović V, Todorović SM, Mennerick S, Powell S, Dikranian K, Benshoff N (1998). Nitrous oxide (laughing gas) is an NMDA antagonist, neuroprotectant and neurotoxin. Nat Med.

[26] Finck AD, Samaniego E, Ngai SH (1995). Nitrous oxide selectively releases met5-enkephalin and met5-enkephalin-arg6-phe7 into canine third ventricular cerebrospinal fluid. Anesth Analg.

[27] Huang Y, Tang C, Du J, Jin H (2016). Endogenous sulfur dioxide: a new member of gasotransmitter family in the cardiovascular system. Oxid Med Cell Longev.

[28] Li B, Gao MX, Yang WL, Chai C, Zhang DX, Cai HY (2019). Inhibitory effects of sulfur dioxide within the nucleus tractus solitarii of rats: involvement of calcium ion channels, adenine nucleoside triphosphate-sensitive potassium channels, and the nitric oxide/cyclic guanine trinucleotide phosphate pathway. NeuroReport.

[29] Stimac R, Kerek F, Apell HJ (2003). Macrocyclic carbon suboxide oligomers as potent inhibitors of the Na,K-ATPase. Ann N Y Acad Sci.

[30] Kerek F, Stimac R, Apell HJ, Freudenmann F, Moroder L (2002). Characterization of the macrocyclic carbon suboxide factors as potent Na, K-ATPase and SR Ca-ATPase inhibitors. Biochim Biophys Acta Biomembr.

[31] Borowitz JL, Gunasekar PG, Isom GE (1997). Hydrogen cyanide generation by mu-opiate receptor activation: possible neuromodulatory role of endogenous cyanide. Brain Res.

[32] Argyropoulos SV, Nutt DJ (1999). The use of benzodiazepines in anxiety and other disorders. Eur Neuropsychopharmacol.

[33] Carl E, Witcraft SM, Kauffman BY, Gillespie EM, Becker ES, Cuijpers P (2020). Psychological and pharmacological treatments for generalized anxiety disorder (GAD): a meta-analysis of randomized controlled trials. Cogn Behav Ther.

[34] Thibaut F (2017). Anxiety disorders: a review of current literature. Dialogues Clin Neurosci.

[35] Reif A, Herterich S, Strobel A, Ehlis AC, Saur D, Jacob CP (2006). A neuronal nitric oxide synthase (NOS-I) haplotype associated with schizophrenia modifies prefrontal cortex function. Mol Psychiatry.

[36] Reif A, Jacob CP, Rujescu D, Herterich S, Lang S, Gutknecht L (2009). Influence of functional variant of neuronal nitric oxide synthase on impulsive behaviors in humans. Arch Gen Psychiatry.

[37] Wegener G, Volke V (2010). Nitric oxide synthase inhibitors as antidepressants. Pharmaceuticals (Basel).

[38] Pitsikas N (2018). The role of nitric oxide (NO) donors in anxiety. Lights and shadows. Nitric Oxide.

[39] Carreño-Gutiérrez H, O'Leary A, Freudenberg F, Fedele G, Wilkinson R, Markham E (2020). Nitric oxide interacts with monoamine oxidase to modulate aggression and anxiety-like behavior. Eur Neuropsychopharmacol.

[40] Philippu A (2016). Nitric oxide: a universal modulator of brain function. Curr Med Chem.

[41] Zhu LJ, Shi HJ, Chang L, Zhang CC, Si M, Li N (2020). nNOS-CAPON blockers produce anxiolytic effects by promoting synaptogenesis in chronic stress-induced animal models of anxiety. Br J Pharmacol.

[42] Montfort WR, Wales JA, Weichsel A (2017). Structure and activation of soluble guanylyl cyclase, the nitric oxide sensor. Antioxid Redox Signal.

[43] Picón-Pagès P, Garcia-Buendia J, Muñoz FJ (2019). Functions and dysfunctions of nitric oxide in brain. Biochim Biophys Acta Mol Basis Dis.

[44] Edwards TM, Rickard NS (2007). New perspectives on the mechanisms through which nitric oxide may affect learning and memory processes. Neurosci Biobehav Rev.

[45] Heinrich TA, Da Silva RS, Miranda KM, Switzer CH, Wink DA, Fukuto JM (2013). Biological nitric oxide signaling: chemistry and terminology. Br J Pharmacol.

[46] Tomiga Y, Sakai K, Nakashima S, Uehara Y, Kawanaka K, Higaki Y (2020). Effects of inosine monophosphate and exercise training on neuronal nitric oxide synthase in the mouse brain. Neurosci Lett.

[47] Hibbs JB, Taintor RR, Vavrin Z, Rachlin EM (1988). Nitric oxide: a cytotoxic activated macrophage effector molecule. Biochem Biophys Res Commun.

[48] Calabrese V, Mancuso C, Calvani M, Rizzarelli E, Butterfield DA, Stella AM (2007). Nitric oxide in the central nervous system: neuroprotection versus neurotoxicity. Nat Rev Neurosci.

[49] Martínez-Lazcano JC, González-Guevara E, Custodio V, Pérez-Severiano F, Olvera-Pérez K, Salgado-Mozo S (2018). Activity of nitric oxide synthase isoforms in acute brain oxidative damage induced by ozone exposure. Nitric Oxide.

[50] Watkinson WP, Campen MJ, Nolan JP, Costa DL (2001). Cardiovascular and systemic responses to inhaled pollutants in rodents: effects of ozone and particulate matter. Environ Health Perspect.

[51] Ridderbusch IC, Yang Y, Weber H, Reif A, Herterich S, Ströhle A (2020). Neural correlates of NOS1 ex1f-VNTR allelic variation in panic disorder and agoraphobia during fear conditioning and extinction in fMRI. Neuroimage Clin.

[52] Kelm M (1999). Nitric oxide metabolism and breakdown. Biochim Biophys Acta.

[53] Buca BR, Mititelu-Tarţau L, Lupuşoru RV, Popa GE, Rezuş C, Lupuşoru CE (2016). New nitric oxide donors with therapeutic potential. Rev Med Chir Soc Med Nat Iasi.

[54] Seabra AB, Duran N (2017). Nanoparticulated nitric oxide donors and their biomedical applications. Mini Rev Med Chem.

[55] Smriga M, Torii K (2003). Prolonged treatment with l-lysine and l-arginine reduces stress-induced anxiety in an elevated plus maze. Nutr Neurosci.

[56] Srinongkote S, Smriga M, Nakagawa K, Toride Y (2003). A diet fortified with l-lysine and l-arginine reduces plasma cortisol and blocks anxiogenic response to transportation in pigs. Nutr Neurosci.

[57] Smriga M, Ando T, Akutsu M, Furukawa Y, Miwa K, Morinaga Y (2007). Oral treatment with l-lysine and l-arginine reduces anxiety and basal cortisol levels in healthy humans. Biomed Res.

[58] Jezova D, Makatsori A, Smriga M, Morinaga Y, Duncko R (2005). Subchronic treatment with amino acid mixture of l-lysine and l-arginine modifies neuroendocrine activation during psychosocial stress in subjects with high trait anxiety. Nutr Neurosci.

[59] Volke V, Wegener G, Vasar E (2003). Augmentation of the NO-cGMP cascade induces anxiogenic-like effect in mice. J Physiol Pharmacol.

[60] Faria MP, Miguel TT, Gomes KS, Nunes-de-Souza RL (2016). Anxiety-like responses induced by nitric oxide within the BNST in mice: role of CRF1 and NMDA receptors. Horm Behav.

[61] Costa NS, Vicente MA, Cipriano AC, Miguel TT, Nunes-de-Souza RL (2016). Functional lateralization of the medial prefrontal cortex in the modulation of anxiety in mice: left or right?. Neuropharmacology.

[62] Kalouda T, Pitsikas N (2015). The nitric oxide donor molsidomine induces anxiolytic-like behavior in two different rat models of anxiety. Pharmacol Biochem Behav.

[63] Li S, Quock RM (2001). Comparison of N_2_O^−^ and chlordiazepoxide-induced behaviors in the light/dark exploration test. Pharmacol Biochem Behav.

[64] Orfanidou MA, Lafioniatis A, Trevlopoulou A, Touzlatzi N, Pitsikas N (2017). Acute and repeated exposure with the nitric oxide (NO) donor sodium nitroprusside (SNP) differentially modulate responses in a rat model of anxiety. Nitric Oxide.

[65] Papageorgoulis A, Fallon P, Mpalantes N, Papageorgouli D, Pitsikas N (2020). Repeated but not acute exposure with a low dose range of the nitric oxide (NO) donor sodium nitroprusside (SNP) induces anxiolytic-like behavior in a dose-independent manner in two different rat models of anxiety. Nitric Oxide.

[66] Umathe SN, Bhutada PS, Jain S, Munhada YR, Borkar SS, Dhumal B (2009). Role of nitric oxide in obsessive–compulsive behavior and its involvement in the anticompulsive effect of paroxetine in mice. Nitric Oxide.

[67] Trabace L, Kendrick KM (2000). Nitric oxide can differentially modulate striatal neurotransmitter concentrations via soluble guanylate cyclase and peroxynitrite formation. J Neurochem.

[68] Guimarães FS, Beijamini V, Moreira FA, Aguiar DC, de Lucca AC (2005). Role of nitric oxide in brain regions related to defensive reactions. Neurosci Biobehav Rev.

[69] Heiberg IL, Wegener G, Rosenberg R (2002). Reduction of cGMP and nitric oxide has antidepressant-like effects in the forced swimming test in rats. Behav Brain Res.

[70] Kaster MP, Ferreira PK, Santos AR, Rodrigues AL (2005). Effects of potassium channel inhibitors in the forced swimming test: possible involvement of l-arginine-nitric oxide-soluble guanylate cyclase pathway. Behav Brain Res.

[71] Liu F, Yang X, Ma J, Yang Y, Xie C, Tuerhong M (2017). Nitric oxide inhibitory daphnane diterpenoids as potential anti-neuroinflammatory agents for AD from the twigs of Trigonostemon thyrsoideus. Bioorg Chem.

[72] Karolewicz B, Paul IA, Antkiewicz-Michaluk L (2001). Effect of NOS inhibitor on forced swim test and neurotransmitters turnover in the mouse brain. Pol J Pharmacol.

[73] Zhang J, Huang XY, Ye ML, Luo CX, Wu HY, Hu Y (2010). Neuronal nitric oxide synthase alteration accounts for the role of 5-HT1A receptor in modulating anxiety-related behaviors. J Neurosci.

[74] Beheshti F, Hashemzehi M, Hosseini M, Marefati N, Memarpour S (2020). Inducible nitric oxide synthase plays a role in depression- and anxiety-like behaviors chronically induced by lipopolysaccharide in rats: evidence from inflammationand oxidative stress. Behav Brain Res.

[75] Rivier C (1998). Role of nitric oxide and carbon monoxide in modulating the ACTH response to immune and nonimmune signals. NeuroImmunoModulation.

[76] Turnbull AV, Kim CK, Lee S, Rivier CL (1998). Influence of carbon monoxide, and its interaction with nitric oxide, on the adrenocorticotropin hormone response of the normal rat to a physico-emotional stress. J Neuroendocrinol.

[77] Kim CK, Rivier CL (2000). Nitric oxide and carbon monoxide have a stimulatory role in the hypothalamic-pituitary-adrenal response to physico-emotional stressors in rats. Endocrinology.

[78] Czech DA, Jacobson EB, LeSueur-Reed KT, Kazel MR (2003). Putative anxiety-linked effects of the nitric oxide synthase inhibitor l-NAME in three murine exploratory behavior models. Pharmacol Biochem Behav.

[79] Sevgi S, Ozek M, Eroglu L (2006). l-NAME prevents anxiety-like and depression-like behavior in rats exposed to restraint stress. Methods Find Exp Clin Pharmacol.

[80] Forestiero D, Manfrim CM, Guimarães FS, de Oliveira RM (2006). Anxiolytic-like effects induced by nitric oxide synthase inhibitors microinjected into the medial amygdala of rats. Psychopharmacology.

[81] Gilhotra N, Jain H, Dhingra D (2010). Differential effects of nitric oxide synthase inhibitors on anxiety in unstressed and stressed mice. Indian J Exp Biol.

[82] Bonassoli VT, Contardi EB, Milani H, de Oliveira RM (2013). Effects of nitric oxide synthase inhibition in the dorsolateral periaqueductal gray matter on ethanol withdrawal-induced anxiety-like behavior in rats. Psychopharmacology.

[83] Quock RM, Nguyen E (1992). Possible involvement of nitric oxide in chlordiazepoxide-induced anxiolysis in mice. Life Sci.

[84] Caton PW, Tousman SA, Quock RM (1994). Involvement of nitric oxide in nitrous oxide anxiolysis in the elevated plus-maze. Pharmacol Biochem Behav.

[85] De Oliveira CL, Del Bel EA, Guimaraes FS (1997). Effects of l-NOARG on plus maze performance in rats. Pharmacol Biochem Behav.

[86] Monzon ME, Varas MM, De Barioglio SR (2001). Anxiogenesis induced by nitric oxide synthase inhibition and anxiolytic effect of melanin-concentrating hormone (MCH) in rat brain. Peptides.

[87] Pokk P, Vali M (2002). The effects of the nitric oxide synthase inhibitors on the behavior of small-platform-stressed mice in the plus-maze test. Prog Neuropsychopharmacol Biol Psychiatry.

[88] Joung HY, Jung EY, Kim K, Lee MS, Her S, Shim I (2012). The differential role of NOS inhibitors on stress-induced anxiety and neuroendocrine alterations in the rat. Behav Brain Res.

[89] Piri M, Nasehi M, Asgariyan M, Zarrindast MR (2012). Influence of nitric oxide agents in the dorsal hippocampus of mice on anxiogenic-like effect induced by histamine. Pharmacol Biochem Behav.

[90] Nikkar E, Ghoshooni H, Hadipour MM, Sahraei H (2019). Effect of nitric oxide on basolateral amygdala on persistence of anxiety and depression in stressed male rats. Basic Clin Neurosci.

[91] Vila-Verde C, Marinho AL, Lisboa SF, Guimarães FS (2016). Nitric oxide in the prelimbic medial prefrontal cortex is involved in the anxiogenic-like effect induced by acute restraint stress in rats. Neuroscience.

[92] Spiacci A, Kanamaru F, Guimarães FS, Oliveira RM (2008). Nitric oxide-mediated anxiolytic-like and antidepressant-like effects in animal models of anxiety and depression. Pharmacol Biochem Behav.

[93] Paul BD, Snyder SH (2018). Gasotransmitter hydrogen sulfide signaling in neuronal health and disease. Biochem Pharmacol.

[94] Kumar M, Sandhir R (2018). Hydrogen sulfide in physiological and pathological mechanisms in brain. CNS Neurol Disord Drug Targets.

[95] Park J, Kim T, Kim HJ, Hong J-I (2019). Iridium(iii) complex-based Electrochemiluminescent probe for H_2_S. Dalton Trans.

[96] Bos EM, van Goor H, Joles JA, Whiteman M, Leuvenink HG (2015). Hydrogen sulfide: physiological properties and therapeutic potential in ischaemia. Br J Pharmacol.

[97] Abe K, Kimura H (1996). The possible role of hydrogen sulfide as an endogenous neuromodulator. J Neurosci.

[98] Han Y, Qin J, Chang X, Yang Z, Bu D, Du J (2005). Modulating effect of hydrogen sulfide on gamma-aminobutyric acid B receptor in recurrent febrile seizures in rats. Neurosci Res.

[99] García-Bereguiaín MA, Samhan-Arias AK, Martín-Romero FJ, Gutiérrez-Merino C (2008). Hydrogen sulfide raises cytosolic calcium in neurons through activation of L-type Ca^2+^ channels. Antioxid Redox Signal.

[100] Schreier SM, Muellner MK, Steinkellner H, Hermann M, Esterbauer H, Exner M (2010). Hydrogen sulfide scavenges the cytotoxic lipid oxidation product 4-HNE. Neurotox Res.

[101] Li L, Moore PK (2007). An overview of the biological significance of endogenous gases: new roles for old molecules. Biochem Soc Trans.

[102] Wagner F, Asfar P, Calzia E, Szabo C (2009). Bench-to-bedside review: Hydrogen sulfide—the third gaseous transmitter: applications for critical care. Crit Care (London, England).

[103] Ren C, Du A, Li D, Sui J, Mayhan WG, Zhao H (2010). Dynamic change of hydrogen sulfide during global cerebral ischemia-reperfusion and its effect in rats. Brain Res.

[104] Minamishima S, Bougaki M, Sips PY, Yu JD, Minamishima YA, Elrod JW (2009). Hydrogen sulfide improves survival after cardiac arrest and cardiopulmonary resuscitation via a nitric oxide synthase 3-dependent mechanism in mice. Circulation.

[105] Knapp J, Heinzmann A, Schneider A, Padosch SA, Böttiger BW, Teschendorf P (2011). Hypothermia and neuroprotection by sulfide after cardiac arrest and cardiopulmonary resuscitation. E Resusc.

[106] Lv B, Chen S, Tang C, Jin H, Du J, Huang Y (2020). Hydrogen sulfide and vascular regulation—an update. J Adv Res.

[107] Chen M, Pritchard C, Fortune D, Kodi P, Grados M (2020). Hydrogen sulfide: a target to modulate oxidative stress and neuroplasticity for the treatment of pathological anxiety. Expert Rev Neurother.

[108] Yakovlev AV, Kurmasheva ED, Ishchenko Y, Giniatullin R, Sitdikova GF (2017). Age-dependent, subunit specific action of hydrogen sulfide on GluN1/2A and GluN1/2B NMDA Receptors. Front Cell Neurosci.

[109] Yakovleva O, Bogatova K, Mukhtarova R, Yakovlev A, Shakhmatova V, Gerasimova E (2020). Hydrogen sulfide alleviates anxiety, motor, and cognitive dysfunctions in rats with maternal hyperhomocysteinemia via mitigation of oxidative stress. Biomolecules.

[110] Yang J, Minkler P, Grove D, Wang R, Willard B, Dweik R (2019). Non-enzymatic hydrogen sulfide production from cysteine in blood is catalyzed by iron and Vitamin B6. Commun Biol.

[111] Olson KR (2018). H2S and polysulfide metabolism: conventional and unconventional pathways. Biochem Pharmacol.

[112] Olson KR, Gao Y, Arif F, Arora K, Patel S, DeLeon ER (2018). Metabolism of hydrogen sulfide (H2S) and production of reactive sulfur species (RSS) by superoxide dismutase. Redox Biol.

[113] Chen WL, Xie B, Zhang C, Xu KL, Niu YY, Tang XQ (2013). Antidepressant-like and anxiolytic-like effects of hydrogen sulfide in behavioral models of depression and anxiety. Behav Pharmacol.

[114] Donatti AF, Soriano RN, Leite-Panissi CRA, Branco LGD, de Sousa AS (2017). Anxiolytic-like effect of hydrogen sulfide (H2S) in rats exposed and re-exposed to the elevated plus-maze and open field tests. Neurosci Lett.

[115] Habibitabar E, Moridi H, Shateri H, Karimi SA, Salehi I, Komaki A (2020). Chronic NaHS treatment improves spatial and passive avoidance learning and memory and anxiety-like behavior and decreases oxidative stress in rats fed with a high-fat diet. Brain Res Bull.

[116] Pan X, Zhang Y, Tao S (2015). Effects of Tai Chi exercise on blood pressure and plasma levels of nitric oxide, carbon monoxide and hydrogen sulfide in real-world patients with essential hypertension. Clin Exp Hypertens.

[117] Mustafa AK, Gadalla MM, Snyder SH (2009). Signaling by gasotransmitters. Sci Signal.

[118] Sharma VS, Magde D (1999). Activation of soluble guanylate cyclase by car-bon monoxide and nitric oxide: a mechanistic model. Methods.

[119] Yi L, Morgan JT, Ragsdale SW (2010). Identification of a thiol/disulfide redox switch in the human BK channel that controls its affinity for heme and CO. J Biol Chem.

[120] Brazier SP, Telezhkin V, Mears R, Müller CT, Riccardi D, Kemp PJ (2009). Cysteine residues in the C-terminal tail of the human BK(Ca)alpha subunitare important for channel sensitivity to carbon monoxide. Adv Exp Med Biol.

[121] Liang S, Wang Q, Zhang W, Zhang H, Tan S, Ahmed A, Gu Y (2014). Carbon monoxide inhibits inward rectifier potassium channels in cardiomyocytes. Nat Commun.

[122] Maitlis P, Haynes A, Chiusoli GP, Maitlis P (2006). Chapter 4 syntheses based on carbon monoxide. Metal-catalysis in industrial organicprocesses.

[123] Taskiran D, Kutay FZ, Pogun S (2003). Effect of carbon monoxide on dopamine and glutamate uptake and cGMP levels in rat brain. Neuropsychopharmacology.

[124] Adach W, Olas B (2019). Carbon monoxide and its donors—their implications for medicine. Future Med Chem.

[125] Ismailova A, Kuter D, Bohle DS, Butler IS (2018). An overview of the potential therapeutic applications of CO-releasing molecules. Bioinorg Chem Appl.

[126] Motterlini R, Clark JE, Foresti R, Sarathchandra P, Mann BE, Green CJ (2020). Carbon monoxide-releasing molecules: characterization of biochemical and vascular activities. Circ Res.

[127] Carvalho-Costa PG, Branco LG, Leite-Panissi CR (2016). Activation of locus coeruleus heme oxygenase-carbon monoxide pathway promoted an anxiolytic-like effect in rats. Braz J Med Biol Res.

[128] McCoubrey WK, Huang TJ, Maines MD (1997). Isolation and characterization of a cDNA from the rat brain that encodes hemoprotein heme oxygenase-3. Eur J Biochem.

[129] Neis VB, Rosa PB, Moretti M, Rodrigues ALS (2018). Involvement of heme oxygenase-1 in neuropsychiatric and neurodegenerative diseases. Curr Pharm Des.

[130] Vincent SR, Das S, Maines MD (1994). Brain heme oxygenase isoenzymes and nitric oxide synthase are co-localized in select neurons. Neuroscience.

[131] Verma A, Hirsch DJ, Glatt CE, Ronnett GV, Snyder SH (1993). Carbon monoxide: a putative neural messenger. Science.

[132] Pineda J, Kogan JH, Aghajanian GK (1996). Nitric oxide and carbon monoxide activate locus coeruleus neurons through a cGMP-dependent protein kinase: involvement of a nonselective cationic channel. J Neurosci.

[133] Cazuza RA, Pol O, Ramos C, Leite-Panissi A (2018). Enhanced expression of heme oxygenase-1 in the locus coeruleus can be associated with anxiolytic-like effects. Behav Brain Res.

[134] Li Y, Zhang L-M, Zhang D-X, Zheng W-C, Bai Y (2020). CORM-3 ameliorates neurodegeneration in the amygdala and improves depression- and anxiety-like behavior in a rat model of combined traumatic brain injury and hemorrhagic shock. Neurochem Int.

[135] Tian H (2014). Advances in the study on endogenous sulfur dioxide in the cardiovascular system. Chin Med J.

[136] Ghasemi E, Afkhami Aghda F, Rezvani ME, Shahrokhi Raeini A, Hafizibarjin Z, Zare MF (2020). Effect of endogenous sulfur dioxide on spatial learning and memory and hippocampal damages in the experimental model of chronic cerebral hypoperfusion. J Basic Clin Physiol Pharmacol.

[137] Shi X, Gao Y, Song L, Zhao P, Zhang Y, Ding Y (2020). Sulfur dioxide derivatives produce antidepressant- and anxiolytic-like effects in mice. Neuropharmacology.

[138] Kamat PK, Kalani A, Tyagi N (2015). Role of hydrogen sulfide in brain synaptic remodeling. Methods Enzymol.

